# Gallstone Ileus Treated by Incidental Meckel’s Diverticulectomy

**DOI:** 10.7759/cureus.14078

**Published:** 2021-03-24

**Authors:** Zachary A Koenig, Jason Turner

**Affiliations:** 1 School of Medicine, West Virginia University, Morgantown, USA; 2 Department of Surgery, West Virginia University, Martinsburg, USA

**Keywords:** gallstone ileus, meckel's diverticulum, diverticulectomy

## Abstract

Gallstone ileus is an uncommon cause of intestinal obstruction in the elderly. It is typically recognized on computed tomography by the presence of pneumobilia and a gallstone in the right iliac fossa. Nonetheless, it is important to consider that gallstone ileus may represent the presentation of another pathology rather than an entity on its own. Here, we report successful retrieval of a gallstone that was causing ileus. Intraoperatively, the gallstone was noted lodged in the terminal ileum distal to an incidentally noted Meckel’s diverticulum. The gallstone was milked proximally into the Meckel’s diverticulum and the base was transected. This case illustrates a rare, but unique, surgical technique utilizing a small bowel diverticulum as a vector for stone removal.

## Introduction

Meckel’s diverticulum is a true diverticulum that arises from the antimesenteric surface of the middle-to-distal ileum due to incomplete obliteration of the vitelline duct during the seventh week of gestation. It is the most common malformation of the gastrointestinal tract [1]. The anomaly is known for its “rule of twos,” being present in 2% of the population, presenting before the age of two, being twice as common in men compared to women, and being located two feet from the ileocecal valve [2].

The majority of Meckel’s diverticulum cases are clinically silent, especially in adults. When they do present, the typical signs of Meckel’s diverticulum are nonspecific such as abdominal tenderness and distension. Rare clinical manifestations of Meckel’s diverticulum include ulceration, hemorrhage, intussusception, neoplasm, and obstruction [3,4]. The primary risk factors for developing symptomatic Meckel’s diverticulum include age younger than 50 years, presence of heterotopic mucosa, male sex, and length greater than 2 cm. The presence of two, three, or four of these criteria increases the possibility of symptomatic Meckel’s diverticulum presentation to 25, 42, and 70%, respectively [4].

Adding to the oddity that is Meckel’s diverticulum, gallstone ileus is an unusual cause of intestinal obstruction that occurs secondary to cholelithiasis alongside a biliary-enteric fistula. It presents in less than 0.5% of patients who present with mechanical small bowel obstruction. Gallstone ileus disproportionally affects elderly females [5]. Here, we present a case report describing gallstone ileus that was removed via a Meckel’s diverticulum conduit in an elderly male.

## Case presentation

**Figure 1:**
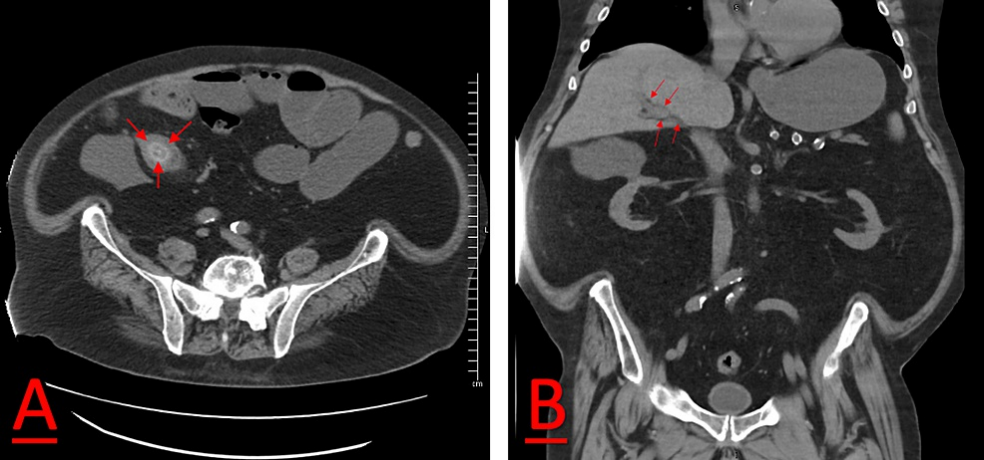
Axial and coronal CT scans demonstrating small bowel obstruction at the terminal ileum. (A) A 1.7 cm gallstone in the terminal ileum, which is causing obstruction, approximately 6 cm from the ileocecal valve (red arrow). (B) The patient’s gallbladder is not clearly seen on imaging, suggesting possible fistula between the gallbladder and the duodenum. There is a small amount of refluxed air on the extrahepatic bile ducts (red arrow). CT, computed tomography

A 78-year-old male with a past medical history of chronic kidney disease, coronary artery disease, atrial fibrillation, congestive heart failure, and obesity presented to his local rural medical center with chief complaints of worsening abdominal pain, bilious vomiting, and lethargy. He was discharged one week prior with an upper gastrointestinal bleed due to a 2 cm duodenal ulcer and was discharged with pantoprazole and sucralfate. However, over the next week, he became unable to walk no more than 15 feet without experiencing excruciating abdominal pain localized to his periumbilical region. During his initial presentation to the emergency department, his abdominal examination demonstrated right lower quadrant tenderness. His emergency department laboratory results were noncontributory. Computed tomography (CT) without contrast showed air in the intrahepatic bile ducts and a radiodense mass in the distal terminal ileum consistent with a stone. There was also proximal dilation of small bowel consistent with obstruction (Figures 1A, 1B).

On the subsequent day, he underwent exploratory laparotomy to remove the gallstone. A midline laparotomy incision was made in the periumbilical region. Sharp dissection was used to carry it down to the level of the peritoneum. Large dilated small bowel loops were immediately encountered, and they were followed to the impacted gallstone which was noted 6 cm proximal to the ileocecal valve. This was elevated into the wound and the stone was easily dislodged from its impacted site. The site where it was lodged was noted to have ecchymosis on the bowel wall with inflammation, but no evidence of perforation or necrosis was noted. Additionally, there was some inflammation in the surrounding mesentery in this region. The gallstone was milked back approximately 20 cm into the dilated proximal portion of the small intestines.

**Figure 2:**
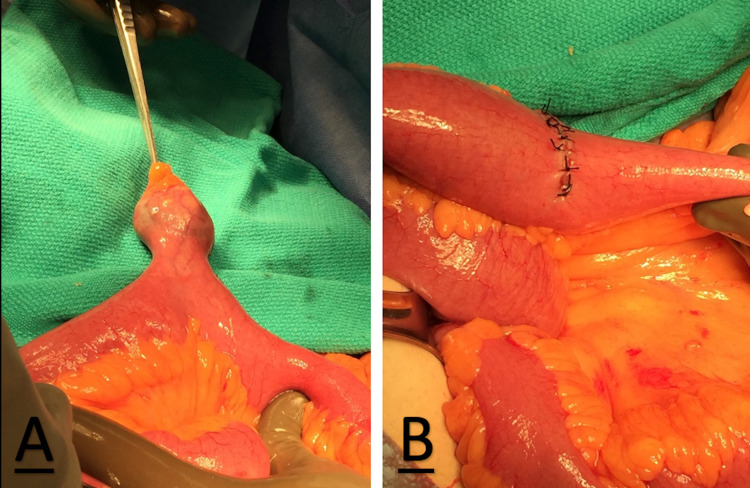
Meckel’s diverticulum site pre- and post-diverticulectomy. (A) The diverticulum had a large broad base and a long stalk. It was found approximately 20 cm proximal to the gallstone impaction. (B) The edges of the colon were easily reattached and sutured together.

**Figure 3:**
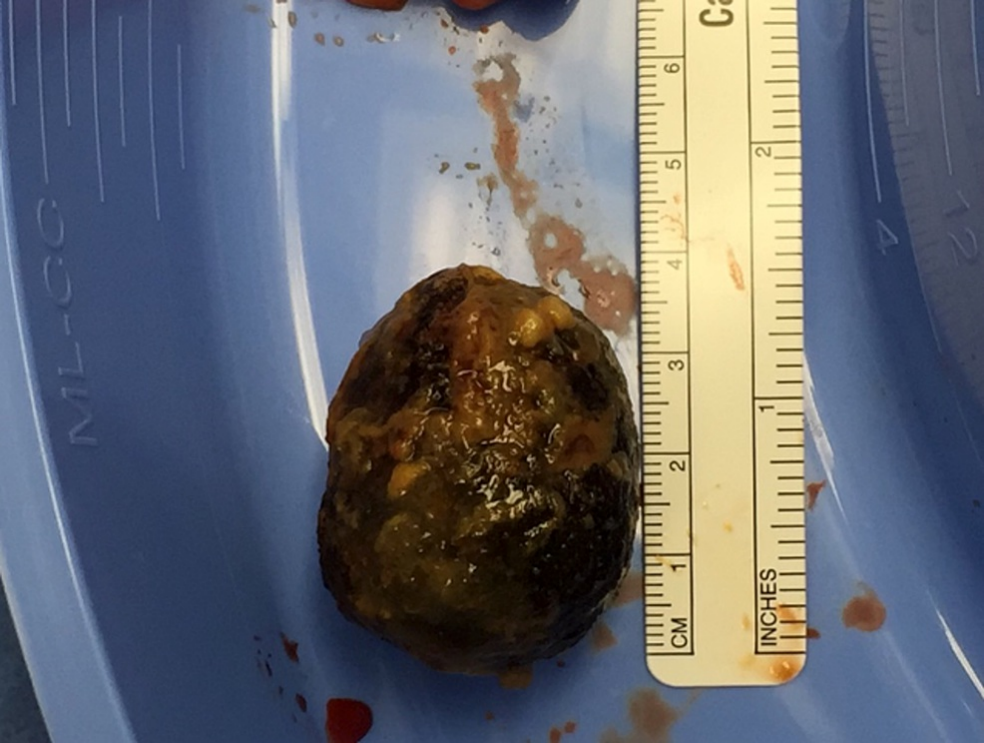
Gross specimen of the gallstone that was impacted in the Meckel’s diverticulum.

At this point, a Meckel’s diverticulum was encountered which had a large broad base and a long stalk (Figure 2A). The gallstone was positioned to be contained within the Meckel’s diverticulum and a thoracoadbominal-55 blue load stapler was used to transect the Meckel’s diverticulum with the gallstone in a horizontal fashion. After this, the staple line was oversewn with 4-0 silk sutures leaving a patent ileal lumen (Figure 2B). The gallstone was then retrieved (Figure 3). Some of the bowel content were milked back into the stomach and removed via nasogastric suction. There was no spillage or contamination from the Meckel’s diverticulectomy. It was decided to withhold cholecystectomy and closure of the fistula due to age and comorbidities.

He was admitted to the intensive care unit where he remained intubated until post-operative day one. Post-operative complications included ileus and acute-on-chronic renal failure, which were managed using intravenous fluids and nasogastric tube placement, respectively. After a few more days of conservative ileus resolution, the nasogastric tube was removed, his renal function improved, and his diet was advanced to solids. He was subsequently discharged after a 10-day hospital stay with complete resolution of his initial symptoms. Five years thereafter, he remains free of abdominal pain and has not experienced any other bouts of gallstone ileus.

## Discussion

Impaction of a gallstone at a Meckel’s diverticulum can cause small bowel obstruction, intermittent abdominal pain, diverticulitis, bleeding, or perforation in severe cases. Localization of stones in a Meckel’s diverticulum makes that area especially vulnerable to obstruction due to diverticular inflammation, diverticular obstruction, or extrusion of the stone from the diverticulum. A diagnostic challenge is differentiation of gallstone ileus and Meckel’s diverticulum enterolith due to similar clinical presentations. Findings that support gallstone ileus include pneumobilia on abdominal film, but it has very low sensitivity for both gallstone ileus and Meckel’s diverticulum enterolith [6]. The ideal imaging modality in this situation is CT which provides sensitivity, specificity, and diagnostic accuracy of over 93% [7]. Nonetheless, preoperative diagnosis of Meckel’s diverticulum is difficult even with imaging because of the rarity of adult cases.

An area of controversy related to this case is the surgical management of an incidentally found Meckel’s diverticulum in the adult population. While most surgeons suggest removal of an asymptomatic diverticulum in the pediatric and young adult population, the literature is scarce regarding prophylactic resection in adults. A study by Peoples et al. noted a high mortality rate associated with resection, but only 6.2% of their patient population developed symptoms from a Meckel’s diverticulum and most of these complications occurred within the first two decades of life [8]. Another study showed that there was a lower operative morbidity and mortality when Meckel’s diverticulum resection was elective (2% and 1%, respectively) as opposed to nonelective (12% and 2%, respectively) [9]. However, Zani et al. recognized that the number of diverticular resections required to prevent death is 758 [10]. Based on these findings, it is not recommended to proceed with prophylactic diverticulectomy in adults. A notable exception to this recommendation is in cases characterized by long length, narrow base, and palpable heterotopic tissue, where operative management is given special consideration.

In contrast to prophylactic Meckel’s diverticulum resection in adults, it is more consistently accepted that complicated and symptomatic Meckel’s diverticulum indicates surgical management. Options for operative management in cases of Meckel’s diverticulum with gallstone impaction include gallstone removal via enterotomy or fragmentation of the gallstone with extorsion into the proximal bowel. Diverticulum resection should also be done to prevent future stone formation and other complications. However, even within this spectrum of management, there is disagreement regarding whether to perform segmental resection versus diverticulectomy. Surgeons can resect the diverticulum easily while simultaneously avoiding entering the bowel lumen using a stapler [11]. Even so, others argue that indications for bowel resection and primary anastomosis include perforation, necrotic bowel, and inflammation. This serves to reduce the risk of bowel stenosing the bowel lumen [12].

Options for operative management of gallstone impaction in the small bowel include simple enterolithotomy, single-stage surgery (enterolithotomy, cholecystectomy, and fistula repair), and two-stage surgery (enterolithotomy with delayed cholecystectomy and fistula closure). This can be done laparoscopically or open, and the benefits of each remain an active area of research. Most surgeons elect for simple enterolithotomy without cholecystectomy and fistula repair due to the low rate of recurrence, low incidence of gallbladder carcinoma, and high mortality rate of multiple procedures at once. Moreover, the majority of fistula close spontaneously after the distal obstruction is relieved [13].

## Conclusions

Meckel’s diverticulum is the most common congenital malformation of the gastrointestinal tract, despite rarely presenting symptomatically in adults. An uncommon presentation of Meckel’s diverticulum arises whenever a gallstone becomes impacted in it, leading to symptoms of bowel obstruction. This creates a diagnostic and therapeutic challenge for surgeons due to intraoperative recognition of the diverticulum. In our case, an obese, high-risk patient benefited from an open approach in which the diverticulum was resected and the stone was removed. After the Meckel’s diverticulum and gallstone ileus have been accounted for, there arises the option for delayed elective cholecystectomy. In patients who develop recurrent gallstone ileus, elective cholecystectomy can be given stronger consideration.
